# Preparation of Temozolomide-Loaded Nanoparticles for Glioblastoma Multiforme Targeting—Ideal Versus Reality

**DOI:** 10.3390/ph9030054

**Published:** 2016-09-08

**Authors:** Chooi Yeng Lee, Ing Hong Ooi

**Affiliations:** 1 School of Pharmacy, Monash University Malaysia, Bandar Sunway, Selangor 47500, Malaysia; 2 Department of Pharmaceutical Chemistry, School of Pharmacy, International Medical University, Kuala Lumpur 57000, Malaysia; inghong_ooi@imu.edu.my

**Keywords:** glioblastoma, polymeric nanoparticles, sustained release, targeted delivery, temozolomide

## Abstract

Temozolomide (TMZ) is one of the most effective chemotherapeutic agents for glioblastoma multiforme, but the required high administration dose is accompanied by side effects. To overcome this problem and to further improve TMZ’s efficacy, targeted delivery of TMZ by using polymeric nanoparticles has been explored. We synthesised the PLGA-PEG-FOL copolymer and attempted encapsulation of TMZ into PLGA-PEG-FOL nanoparticles using the emulsion solvent evaporation method and the nanoprecipitation method. Conjugation of PEG and FOL to PLGA has been reported to be able to increase the delivery of TMZ to the brain as well as targeting the glioma cells. However, despite making numerous modifications to these methods, the loading of TMZ in the nanoparticles only ranged between 0.2% and 2%, and the nanoparticles were between 400 nm and 600 nm in size after freeze-drying. We proceed with determining the release profile of TMZ in phosphate buffered saline (PBS). Our initial data indicated that TMZ was slowly released from the nanoparticles. The metabolite of TMZ rather than the parent compound was detected in PBS. Our study suggests that while PLGA-PEG-FOL can be used as a polymeric or encapsulation material for central delivery of TMZ, a practical and cost effective formulation method is still far from reach.

## 1. Introduction

Glioblastoma multiforme (GBM) is the most common primary brain tumour. It is classified by the World Health Organisation as a grade IV tumour. The current standard treatment for GBM begins with a maximal safe surgical resection, followed by a combined radiotherapy and temozolomide (Temodal^®^, TMZ), and adjuvant TMZ therapy. The treatment regimen is by far the most effective, as it increases the median overall survival to 14.6 months. The percentage of patients alive at two years has also increased from 10.4% to 26.5% [1].

Temozolomide is a second-generation imidazotetrazine pro-drug, which has been shown in various studies to be an effective alkylating agent [2,3]. At physiological pH, TMZ is metabolised to its active metabolite 5-(3-methyl-1-triazenyl)-imidazole-4-carboxamide (MTIC) [4]. Although TMZ has almost 100% bioavailability, due to its short half-life, high dose administration of TMZ is required and this leads to several dose-limiting side effects [3,5,6]. As a means to improve the efficacy of TMZ and lower the risk of side effects, targeted delivery such as the use of nanoparticles has been recommended [5,7].

We used poly(lactic-co-glycolic acid) (PLGA) as our core material in nanoparticle formulation. PLGA is biodegradable and its hydrolytic products, lactic acid and glycolic acid, are effectively eliminated from the body as carbon dioxide and water [8,9]. We attempted to further increase the specificity of the nanoparticles by functionalising the nanoparticles with poly(ethylene) glycol (PEG) and folic acid (FOL). PEG increases the blood residence time of nanoparticles [10], while FOL has high affinity and specificity towards folate receptors [11]. Over-expression of folate receptors is often found on tumour cells, including glioma [12]. Cellular uptake of polymeric nanoparticles conjugated with FOL is 6.7 to 20 times higher than that of non-conjugated nanoparticles [13,14]. The fabrication and the use of PLGA-PEG-FOL as a carrier for anti-cancer drug is not new [15]. We successfully synthesised the PLGA-PEG-FOL copolymer by following the reported procedures with slight modification, and used the copolymer to encapsulate TMZ forming TMZ-loaded nanoparticles. The study investigated various conditions of two commonly used methods in the preparation of TMZ-loaded FOL-linked, PEGylated-PLGA nanoparticles. We then determined the percentage of TMZ loading in the nanoparticles and the size of the nanoparticles.

## 2. Results

The formulation and characteristics of TMZ-loaded PLGA-PEG-FOL nanoparticles prepared using the emulsion solvent evaporation method (Method 1) are shown in Table 1. In this method, the use of DCM as the organic phase yielded TMZ-loaded PLGA-PEG-FOL nanoparticles with a DL of 1%, and the particle size was approximately 420 nm. Increasing the amount of TMZ to 2.5 mg from 2 mg reduced the DL tremendously by 10-fold. Suspending 5 mg of TMZ in 2 mL of DCM increased the DL to 2%, but the yield of the formulation was only 40% (Table 1). From the second solvent evaporation method, the DL was only 0.2%. However, this method increased the yield to 68%, and the particle size was reduced to less than 400 nm (Table 2). It should be noted that the sizes of the nanoparticles presented in Table 1 and Table 2 were measured after freeze-drying.

Table 3 is the summary of the formulation and characteristics of TMZ-loaded PLGA-PEG-FOL nanoparticles prepared using emulsion solvent evaporation involving dual solvent and two-phase TPGS solution (Method 3).

In this method, DMSO/DCM and DMF/DCM mixed solvents were used as the dispersed phases of the drug and copolymer, and the two-phase dispersing phases contained TPGA that acted as the surfactant. The DL remained low at 0.2%, and the particle sizes was approximately 470 nm (preparation 1, Table 3). Substituting DMSO with DMF and saturating the aqueous phase with sodium chloride did not improve the DL (Table 3). On the other hand, TMZ-loaded PLGA-PEG-FOL nanoparticles preparation using the nanoprecipitation method (Table 4) has resulted in 10 to 100 times reduction in the TMZ loading compared with that observed from the solvent evaporation methods. Besides that, there was an increase in the size of the TMZ-loaded nanoparticles. Nonetheless, saturating the external phase with sodium chloride improved the yield, EE, DL and the size of the nanoparticles (preparations 5 vs. 7, Table 4). In all the modified solvent emulsification methods and the nanoprecipitation method, the nanoparticle size increased tremendously after freeze-drying, as indicated by their size measured using the Zetasizer (Table 3 and Table 4).

**Figure 1:**
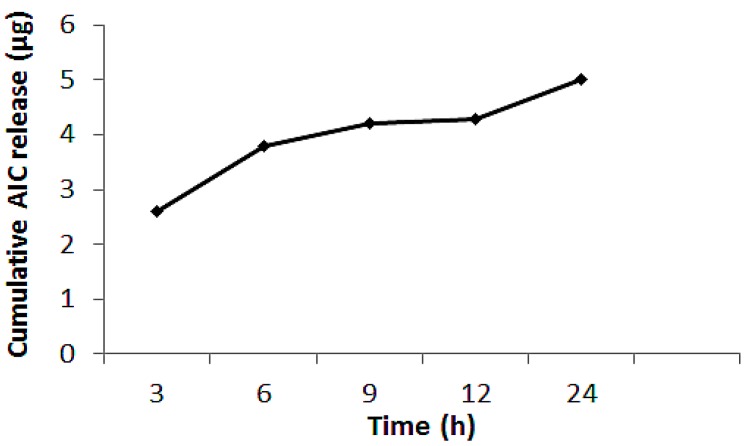
The release of the metabolic product of TMZ, 5-aminoimidazole-4-carboxamide (AIC) in vitro from the TMZ-loaded PLGA-PEG-FOL nanoparticles.

In the in vitro release study, we did not detect TMZ in the supernatant. But a peak, which was consistent to that of the AIC was detected, and the peak size increased gradually between 3 h and 24 h. At 3 h, the amount of AIC was 2.6 μg and the amount doubled to 5 μg only after 24 h (Figure 1). The AIC amount was equivalent to 8.5% and 16.7% of the amount of the TMZ used.

## 3. Discussion

Data from the first solvent evaporation method (from preparation 3, Table 1) suggested that when DCM was used as an organic solvent, high TMZ concentration may be required to achieve a better DL. At higher TMZ concentration, less drug is expected to diffuse out through the organic-aqueous interphase, i.e., from the organic phase into the dispersing phase. Ling et al. [16] showed that the use of DMF as an organic solvent and TPGS as the surfactant give a DL of between 0.15% and 17.95%, and the DL increases in proportion to the amount of TMZ used, which is at the range between 10 mg and 80 mg. DMF is a polar solvent that is able to dissolve TMZ, and TPGS is a derivative of vitamin E, thus is water soluble. TPGS is reported to have 67 times higher emulsification effects than PVA on PLGA-based nanoparticles [17,18]. However, residual PVA that clung to the nanoparticles has prevented dissolving of the nanoparticles for direct measurement of the DL using UV/Vis spectrophotometry technique. Therefore, in our subsequent preparation (Table 2), we increased the amount of TMZ by 10-fold (vs. preparation 1, Table 1) and substituted DCM with DMF, and replaced PVA with TPGS. However, our results were inconsistent with those reported previously [16].

From the solvent evaporation method, other study has shown that having 20 mg of TMZ in the organic phase, and saturating the aqueous dispersing phase with TMZ gives a higher TMZ loading than that of an dispersing phase not saturated with TMZ (14% vs. 0.4%) [16]. However, this approach means that a large amount of TMZ will be required in preparing the nanoparticles, and this implies high cost and waste. We attempted a modified saturation method [19,20]. In this modified method, an emulsion was first generated when the organic phase was added to a smaller volume of the TPGS solution, and this was aimed to minimise the diffusion of TMZ under shear force during the second emulsification, leveraging also on the TPGS concentration gradient. By using a higher amount of TMZ, it may saturate the first TPGS solution when the primary emulsion is generated, hence may improve drug entrapment. The primary emulsion is then added to a larger volume of TPGS solution under stirring to encourage diffusion and evaporation of the organic solvent. We used solvent to dissolve or suspend TMZ as the drug has limited solubility in DCM. The ratios of the co-solvent used, as shown in Table 3, were the optimal combination.

Besides that, in preparation 3 (Table 3), the dispersing phase was comprised of TPGS dissolved in ethanol/water mixture saturated with sodium chloride. Because TMZ has lower solubility in ethanol than in water, we estimated that ethanol may reduce the diffusion of TMZ to the TPGS aqueous solution, and therefore give better entrapment of TMZ in the nanoparticles. Sodium chloride was used to saturate the TPGS solution so as to reduce the concentration gradient of the solute between the solvent and the aqueous phase, thereby reducing the diffusion potential and improves the encapsulation of TMZ [21,22]. Nonetheless, ethanol and sodium chloride have little effect on the loading efficiency as the preparation has comparably low DL as with other preparations (Table 3). It is unclear why sodium chloride did not improve the loading. It is worth noting that from preparations 1 to 3, the primary emulsion formed large emulsion droplets when it was introduced to the subsequent TPGS solution, and the large emulsion droplets could only be dispersed with shear forces [23].

Nanoprecipitation method is widely used in studies involving hydrophilic drugs [21,24,25]. However, in this study, using either water or ethanol only as a dispersing phase did not improve the DL of TMZ (Table 4). A combination of ethanol and water with a ratio of 85 to 15, and saturation of the mixture with sodium chloride led to an increase in EE and DL. Saturated ethanol and water solution, with ethanol and water prepared at different ratios did not give such effects. Overall, nanoprecipitation method did not compare favorably against solvent evaporation method in our preparation of TMZ loaded PLGA-PEG-FOL nanoparticles.

It was noted that, in all the solvent emulsification and nanoprecipitation methods studied, the nanoparticle size increased significantly to between 370–520 nm (Table 3) and 430–630 nm (Table 4) after freeze-drying. This was most likely due to particle aggregation under cryogenic stress condition. Cryo-protectant will be needed to minimise the particle agglomeration effect. In contrast, nanoparticles that were not subjected to free-drying cycle were generally less than 200 nm.

In the in vitro release study, there are two main reasons as to why TMZ was not detected in the PBS–(1) TMZ is hydrolytically labile and most of the TMZ may have already been degraded the moment it was released to the medium; (2) Due to its degradation, TMZ, if present, has amount that was too little to be detected in the medium. Despite this, the cumulative profile of AIC suggested a sustained release of TMZ from the PLGA-PEG-FOL nanoparticles. This property of the nanoparticles is beneficial as it reduces the needs of frequent administration.

## 4. Materials and Methods

### 4.1. General Information

PLGA (molecular weight of 50,000 Dalton) was purchased from JenKem Technology (Plano, TX, USA), and PEG (molecular weight of 3500 Dalton) was supplied by DURECT Corporation (Birmingham, AL, USA). All the other chemicals and reagents were from Sigma (St. Louis, MO, USA). The solvents used were of analytical grade. NMR spectra were recorded on a Bruker 600 MHz HNMR spectrometer in the University of Malaya, Kuala Lumpur. Deuterated dimethyl sulfoxide (DMSO-d6) was used as a solvent with tetramethysilane as an internal standard. Double beam UV-visible spectrophotometer (Perkin Elmer Lambda 25), a UV-Vis spectroscopy technique, was used to measure the drug loading and drug encapsulation efficiency, and in the drug release studies.

### 4.2. Preparation of PLGA-PEG-COOH

PLGA-PEG was synthesised using a reported method [26]. Briefly, *N*-hydroxysuccinimide (NHS, 3 mg, 0.026 mmol) and 1-ethyl-3-(3-dimethylaminopropyl)carbodiimide hydrochloride (EDC, 5 mg, 0.026 mmol) were dissolved in dichloromethane (DCM, 2 mL), followed by addition of PLGA-COOH (250 mg, 0.005 mmol) dissolved in DCM (2 mL) with stirring. The resulting solution was then precipitated with diethyl ether–methanol (20 mL, 50:50, *v*/*v*%), followed by centrifugation at 8000 *g* for 20 min to remove residual NHS and EDC. Centrifugation and washing with deionised water were repeated twice. The PLGA-NHS pellet was obtained and dried under vacuum to remove residual diethyl ether and methanol.

**Scheme 1:**
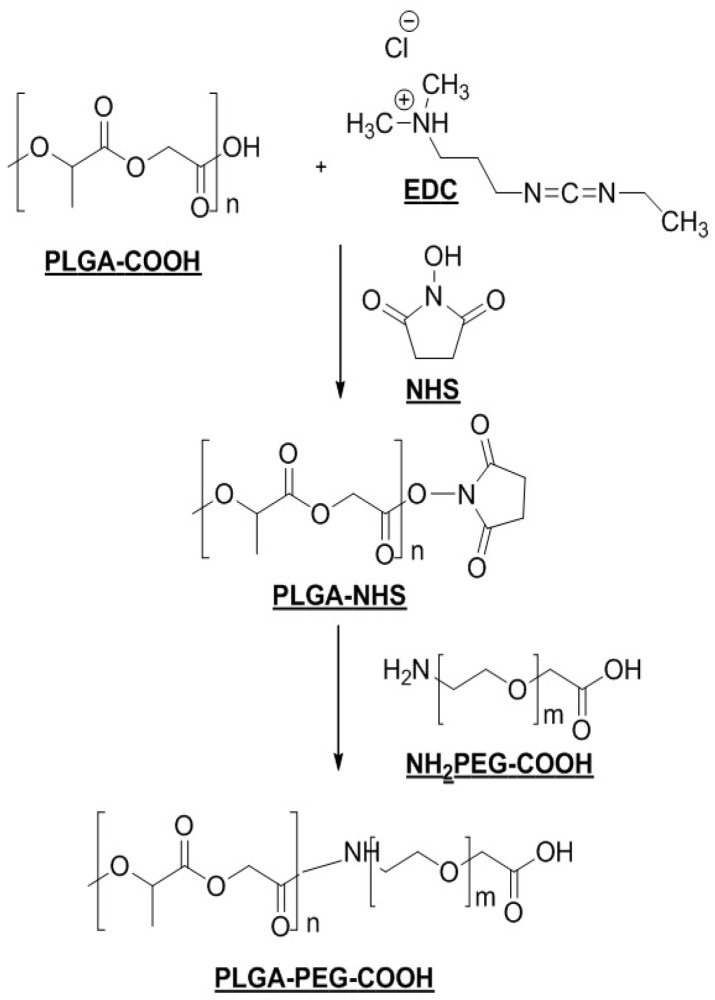
Reaction scheme for the synthesis of PLGA-PEG-COOH from PLGA-COOH.

To prepare PLGA-PEG-COOH (Scheme 1), NH_2_-PEG-COOH (20.4 mg, 0.006 mmol) and *N*,*N*-diisopropylethylamine (DIEA, 7.5 mg, 0.06 mmol) were added to a solution of PLGA-NHS (200 mg, 0.004 mmol) in DCM (4 mL) and then stirred for 24 h at room temperature. The resulting solution was precipitated with diethyl ether–methanol (20 mL, 1:1; *v*/*v*%) solvent mixture and centrifuged at 8000 *g* for 20 min to remove the unreacted PEG and DIEA. Centrifugation and washing with deionised water were repeated twice. The PLGA-PEG-COOH pellet was thus obtained and dried under vacuum.

NHS (2.3 mg, 0.02 mmol) and EDC (4.0 mg, 0.02 mmol) were dissolved in DCM (2 mL) and then added to PLGA-PEG-COOH (200 mg, 0.0037 mmol) solution in DCM (4 mL) with stirring for 60 min at room temperature. The resulting solution was precipitated with excess cold methanol and centrifuged at 8000 *g* for 20 min to remove the excess NHS and EDC. Centrifugation and washing were repeated twice. The PLGA-PEG-NHS obtained was dried under vacuum.

**Scheme 2:**
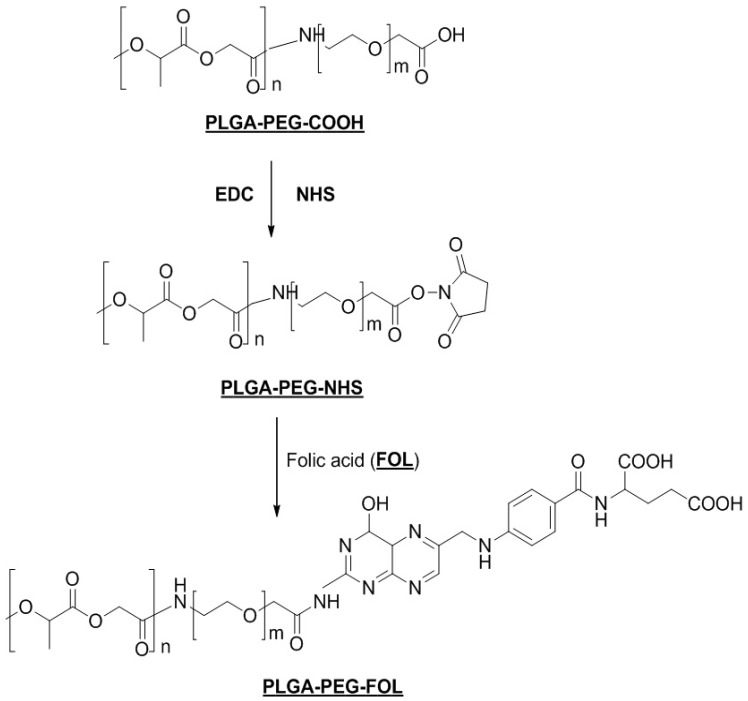
Reaction scheme for the synthesis of PLGA-PEG-FOL.

**Figure 2:**
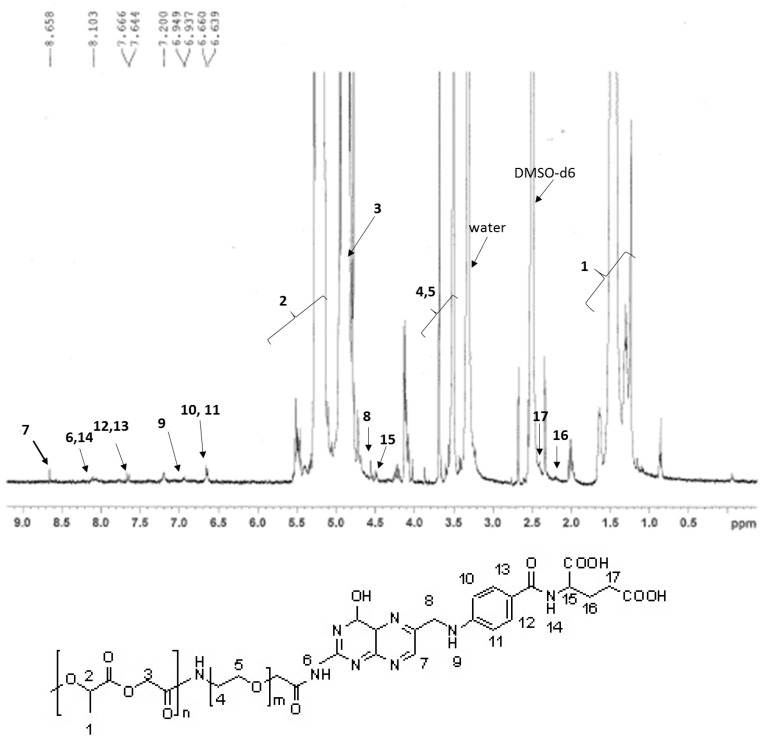
(**Top**) The NMR spectrum of PLGA-PEG-FOL. (**Bottom**) The numbering in the chemical structure of PLGA-PEG-FOL refers to the corresponding proton resonance peaks in the NMR spectrum.

Folic acid was conjugated to PLGA-PEG-NHS by an amidation reaction (Scheme 2). In this preparation, PLGA-PEG-NHS (200 mg, 0.0037 mmol) was dissolved in dimethyl sulfoxide (DMSO, 4 mL). Folic acid (5–10 molar in excess) was added into the solution and stirred in the dark for 24 h at room temperature. The solution was precipitated with cold methanol (20 mL) and centrifuged at 8000 *g*g for 20 min. The precipitate, PLGA-PEG-FOL, was collected, dried under vacuum and then dissolved in DMSO (5 mL) before it was placed in a dialysis bag and dialysed for 24 h against deionised water to remove unreacted folic acid. The cut-off molecular weight of the dialysis membrane was 11,295 Dalton. PLGA-PEG-FOL was characterised by ^1^H-NMR (DMSO-d6, ppm: 1.55, 3.41, 4.71–5.01, 6.64, 6.90, 7.65, 7.87). Figure 2 shows the HNMR spectrum which confirmed the chemical structure of PLGA-PEG-FOL.

### 4.3. Preparation of TMZ Loaded Nanoparticles by Using Emulsion Solvent Evaporation Method (1)

Two milligrams of TMZ were dissolved in DCM (4 mL), and the solution was homogenised by sonication before addition of PLGA-PEG-FOL copolymer (50 mg). The solution was then added slowly into 1% (*w*/*v*) polyvinyl alcohol (PVA, 20 mL) aqueous solution and probe sonicated at 50 W for 1 min in an ice bath. The emulsion was stirred for 2 h and ultra-centrifuged at 10,000 rpm for 30 min. The supernatant was removed after each centrifugation and the pellet, i.e., nanoparticles were washed three times with deionised water before being freeze-dried. The amount of TMZ encapsulated in the nanoparticles was calculated as the difference between the initial TMZ amount used and the TMZ recovered from the supernatant [27]. The amount of TMZ was quantified by UV/Vis spectrophotometry technique measured at 330 nm, and drug loading (DL) was defined as the percentage of drug mass in the nanoparticles. Encapsulation efficiency (EE) was calculated as the percentage of actual drug mass encapsulated in the nanoparticles in relative to the initial amount of drug used [16]. The yield of the formulation was determined by calculating the difference between the total mass of nanoparticles after freeze-drying (M2) and the total mass of copolymer and TMZ used in preparing the formulation (M1) and from (M2/M1) × 100%.

### 4.4. Preparation of TMZ Loaded Nanoparticles by Using Emulsion Solvent Evaporation Method (2)

Experiments were conducted according to the procedures reported previously with some modification [16]. TMZ (20 mg) was dissolved in dimethylformamide (DMF, 1 mL) before the addition of copolymer (50 mg). The solution was slowly added into 0.05% (*w*/*v*) D-α-tocopheryl polyethylene glycol 1000 succinate (vitamin E TPGS, 25 mL) aqueous solution and emulsified for 5 min in ice-bath using a micro-tip probe sonicator operated in pulse mode. The oil-in-water emulsion was stirred for 4 h at room temperature to allow diffusion of the organic solvent and solidification of the nanoparticles. The solution was ultra-centrifuged at 10,000 rpm for 20 min and the pellet washed three times with deionised water to remove the un-encapsulated TMZ. Nanoparticles were freeze-dried, and the lyophilised nanoparticles were dissolved in acetonitrile to determine the DL. The EE and the yield were measured as described above.

### 4.5. Preparation of TMZ Loaded Nanoparticles by Using Emulsion Solvent Evaporation Method (3)

The following procedures were modified from methods reported by Italia et al. [19] and Ling et al. [20]. Twenty milligrams of TMZ were dissolved in 2 mL of DMSO–DCM (1:3) or DMF–DCM (0.6:1.4), followed by the addition of PLGA-PEG-FOL copolymer (50 mg). The solution was mixed with aqueous solution containing 0.2% (*w*/*v*) TPGS (2 mL) and emulsified for 5 min using a probe sonicator (output power of 50 W in pulse mode). The emulsion was then added to a second TPGS solution (15 mL, 0.025% (*w*/*v*)) placed under mechanical stirring at 600 rpm, and the oil-in-water emulsion was agitated for 4 h at room temperature. The suspension was centrifuged at 10,000 rpm for 20 min, and the pellet washed with deionised water to remove residual drug and surfactant. The nanoparticles were freeze-dried, dissolved in acetonitrile, and the TMZ loading was determined by UV/Vis spectrophotometry.

### 4.6. Preparation of TMZ Loaded Nanoparticles by Using the Nanoprecipitation Method

According to this method, the polymer and the drug are dissolved in a solvent that is miscible with water. The polymer-drug solution is dispersed into the aqueous dispersing medium in which nanoparticles will be formed immediately when the solvent diffuses into the dispersing phase. Drug is entrapped when the polymer precipitated and solidified into nanoparticles [21,24,25]. Solutions of 15 mL of 1% (*w*/*v*) Poloxamer 188 in ethanol, water, and ethanol/water mixtures with varying proportion as dispersing media were prepared, respectively. Dispersing media saturated with sodium chloride were also prepared and evaluated. TMZ (3 mg) and PLGA-PEG-FOL copolymer (30 mg) were dissolved in DMSO (3 mL) and the DMSO solution was added dropwise (0.3 mL/min) to the dispersing phase using a syringe (with a 25G needle) positioned above the medium, which was under moderate stirring. Stirring was continued for another 2 h to allow diffusion of the organic solvent. The resulting solution was centrifuged at 25,000 rpm for 30 min and the nanoparticles that appeared as the pellet were subjected to freeze-drying. The lyophilised nanoparticles were weighed and dissolved in acetonitrile, and the amount of encapsulated TMZ was determined by UV/Vis spectrophotometry.

### 4.7. Characterisation of the Nanoparticles

The particle size, size distribution and the surface charges (i.e., zeta potentials) of the nanoparticles were measured using a Nano-ZS Zetasizer equipped with a dynamic light scattering detector (Malvern Instruments, Malvern, UK).

### 4.8. In Vitro Release Study

We examined the release profile of TMZ from the nanoparticles that were generated from the solvent evaporation Method 3, as this method gave us a better yield compared to all the other methods. The release study was conducted as reported before with minor modification [16,28]. TMZ is hydrolytically labile, therefore its degradation product 5-aminoimidazole-4-carboxamide (AIC) was also measured [28]. Fifteen milligram of nanoparticles were suspended in phosphate-buffered saline (PBS, pH 7.4, 2 mL) in a screw-capped tube and incubated in an orbital shaker at 37 °C. At the end of each time point (3, 6, 9, 12 and 24 h), the suspension was centrifuged at 10,000 rpm for 10 min. The supernatant was collected and analysed for TMZ and AIC by UV/Vis spectrophotometry at 330 nm and 267 nm, respectively. The absorbance wavelength of the AIC was determined by using the standard compound of AIC. The pellet was re-suspended in 2 mL of fresh PBS and incubation continued.

## 5. Conclusions

The methods used in this study are not new, but the idea of making TMZ loaded into PLGA-PEG-FOL nanoparticles, i.e., the combination of TMZ and PLGA-PEG-FOL has not been reported before. PLGA-PEG-FOL may allow targeted delivery of the drug, and the final product would be ideal for TMZ delivery. However, as the formulation attempts were not successful, we should consider finding a balance between being idealistic in our experimental design and what we can actually achieve in practice.

Our data suggested that neither the emulsion solvent evaporation method nor the nanoprecipitation method is suitable for our intended preparation. Both methods are commonly used in the preparation of polymeric nanoparticles. Despite the numerous and extensive modifications done on these methods, the percentage of drug loading was not satisfactory. Reflecting on the discrepancy between our data and with that reported in the literature, it appears that the amount of TMZ used in preparing TMZ nanoparticles, as well as the solubility of TMZ in the solvent used to dissolve the drug, are critical in achieving a high DL. The saturation method [16] gives a much improved DL, but it is only a 30-fold increase in DL compared to a non-saturated condition. Thus, creating a simulated saturated condition may be a better alternative and is more cost effective.

The technical difficulties encountered in the present study could also be attributed to the loss of drug during the emulsification or precipitation step, where TMZ diffused out into the aqueous dispersing medium under high shear force. Unless these issues are resolved, the production of TMZ-PEG-PLGA-FOL nanoparticles using these two methods remains challenging. A more realistic solution here might be to explore new preparation method to obtain these nanoparticles. Despite having a relatively low DL, the release profile of the PLGA-PEG-FOL nanoparticles suggested that TMZ was released in a sustained manner, and this is indeed a positive outcome achieved in this study.

## Figures and Tables

**Table 1:** The preparation and the properties of TMZ-loaded nanoparticles developed using the solvent evaporation method.

Preparation *	TMZ Amount (mg)	Solvent and Volume	Yield (%)	EE (%)	DL (%)	Particle Size ± SD (nm)	Zeta Potential ± SD (mV)
1 ^	2	4 mL DCM	54.2 ± 4.9	11.90 ± 4.4	1.05 ± 0.5	424 ± 94	−20.8 ± 13.6
2 ^	2.50	4 mL DCM	71.15 ± 7.85	2.59 ± 1.82	0.18 ± 0.11	611 ± 21	−24.1 ± 2.97
3	5	2 mL DCM	39.9	12.60	2.00	416 ± 21	−15.5 ± 5.3

* The organic phase was added to 20 mL of water containing 1% PVA. PLGA-PEG-FOL copolymer was at 50 mg. EE: encapsulation efficiency, DL: drug loading. ^ Experiment was repeated and data were expressed as mean ± SD (*n* = 2).

**Table 2:** The preparation and the property of TMZ-loaded nanoparticles developed using the solvent evaporation method, and with DMF used as the organic phase.

Preparation *	TMZ Amount (mg)	Solvent and Volume	Yield (%)	EE (%)	DL (%)	Particle Size ± SD (nm)	Zeta Potential ± SD (mV)
1 ^	20	1 mL DMF	67.90 ± 17.40	0.29 ± 0.06	0.19 ± 0.18	394 ± 26	−37.3 ± 1.98

* This preparation has 0.05% TPGS added to the 20 mL of the aqueous phase. PLGA-PEG-FOL copolymer was at 50 mg. EE: encapsulation efficiency, DL: drug loading. ^ Experiment was repeated and data were expressed as mean ± SD (*n* = 2).

**Table 3:** The preparation and the properties of TMZ-loaded nanoparticles developed using dual solvent and two-phase TPGS solution.

Preparation *	Solvent (2 mL)	TPGS 1 (2 mL)	TPGS 2 (15 mL)	Yield (%)	EE (%)	DL (%)	Particle Size ± SD (nm) before Freeze-Drying	Particle Size ± SD (nm) after Freeze-Drying	Zeta Potential ± SD (mV)
1	DMSO:DCM 0.5:1.5	0.2% in water	0.025% in water	70.2	0.27	0.19	310.90 ± 9.76	473.10 ± 48.5	−56.76 ± 1.06
2	DMF:DCM 0.6:1.4	0.2% in water	0.025% in water	83	0.26	0.13	146.60 ± 0.14	374.30 ± 14.71	−35.15 ± 0.071
3	DMF:DCM 0.6:1.4	0.2% in ethanol:water (85:15) sat. with NaCI	0.025% in ethanol:water (85:15) sat. with NaCI	88.5	0.32	0.14	157.85 ± 4.17	520.50 ± 21.28	−56.65 ± 0.212

* All preparations used 20 mg TMZ, and 50 mg PLGA-PEG-FOL copolymer. NaCI: sodium chloride.

**Table 4:** The preparation and the properties of TMZ-loaded nanoparticles developed using the nanoprecipitation method.

Preparation *,^	External Phase (15 mL)	Saturation with NaCI	Yield (%)	EE (%)	DL (%)	Particle Size ± SD (nm) before Freeze-Drying	Particle Size ± SD (nm) after Freeze-Drying	Zeta Potential ± SD (mV)
1	Water	No	69.85 ± 1.62	0.32 ± 0.03	0.045 ± 0.01	187.55 ± 0.21	454.75 ± 5.20	−39.6 ± 0.42
2	Ethanol	No	50.70 ± 9.90	0.31 ± 0.12	0.065 ± 0.01	Not measured	501.70 ± 55.58	−35.9 ± 1.84
3	Water	Yes	87.85 ± 3.32	0.35 ± 0.10	0.035 ± 0.01	199.45 ± 0.35	511.50 ± 37.05	−40.0 ± 0.21
4	Ethanol:Water (50:50)	Yes	88.20 ± 2.12	0.19 ± 0.02	0.021 ± 0.001	180.60 ± 12.30	501.20 ± 9.48	−40.2 ± 11.3
5	Ethanol:Water (85:15)	Yes	92.30 ± 0.42	1.15 ± 0.07	0.170 ± 0.01	164.30 ± 3.82	436.50 ± 37.50	−47.0 ± 2.69
6	Ethanol:Water (95:5)	Yes	58.35 ± 7.14	0.39 ± 0.06	0.075 ± 0.01	150.45 ± 2.62	632.5 ± 58.69	−47.3 ± 6.29
7	Ethanol:Water (85:15)	No	47.20 ± 0.99	0.27 ± 0.03	0.060 ± 0.01	156.50 ± 0.28	517.65 ± 13.79	−51.2 ± 1.41

* Each preparation suspended 3 mg of TMZ and 30 mg of copolymer in 3 mL of DMSO, which acted as the organic phase. The external phase contained 1% (*w*/*v*) Poloxamer 188. ^ Each experiment was repeated, and the data were expressed as mean ± SD (*n* = 2).
